# Effects of Abiraterone Acetate on Chronic Kidney Disease in 2 Patients With Metastatic Castration-resistant Prostate Cancer

**DOI:** 10.1097/MD.0000000000000163

**Published:** 2014-12-12

**Authors:** Edoardo Francini, Roberto Petrioli, Anna Ida Fiaschi, Letizia Laera, Giandomenico Roviello

**Affiliations:** From the Medical Oncology Unit, Policlinico Umberto I Hospital, University of Rome, Roma (EF); Pharmacology Unit (AIF); and Medical Oncology Unit, University of Siena, Siena, Italy (RP, LL, GR).

## Abstract

Prostate cancer is the second leading cause of cancer-related death in men in most Western countries. In this report, we present 2 cases of metastatic castration-resistant prostate cancer and chronic kidney disease. Both patients underwent and developed clinical resistance to androgen-deprivation therapy. Subsequently, the patients were treated with the conventional chemotherapeutic approach, which resulted in the worsening of renal function and performance status. Therefore, we opted for treatment with abiraterone acetate, and the patients exhibited improvements in renal function with good response of the disease.

## INTRODUCTION

Abiraterone acetate (AA) is a novel irreversible inhibitor of CYP17 that was approved by the Food and Drug Administration (FDA) on April 28, 2011 in combination with prednisone for the treatment of metastatic castration-resistant prostate cancer (mCRPC), which was previously treated with docetaxel. The approval of this agent was based on a multinational randomized, double-blind, placebo-controlled, phase III trial that involved 1195 patients with docetaxel-refractory CRPC.^1^ This study demonstrated a median overall survival in the AA arm of 14.8 months compared with 10.9 months in the placebo arm (hazard ratio: 0.646, 95% confidence interval: 0.54–0.77; *P* = 0.0001). Furthermore, in 2013, AA was approved by regulatory agencies in the United States and Europe for chemotherapy-naive mCRPC. This approval followed positive results from a phase III COU-AA-302 trial in which 1088 chemotherapy-naive men with metastatic CRPC who were treated with AA/prednisone exhibited prolonged overall survival compared with those treated with a regimen of placebo/prednisone.^2^ More recently, the prespecified interim analysis of the efficacy and safety outcomes in the COU-AA-302 study revealed median overall survivals of 35.3 months on AA versus 30.1 months on placebo.^3^

Within the histories of patients with prostate cancer, the development of a chronic kidney disease (CKD) is not uncommon. In this report, we describe 2 patients with mCRPC and CKD who were treated with AA and experienced improvements in renal function, dramatic declines in prostate-specific antigen (PSA) levels, improvements in performance status, and reductions in bone pain.

## CASE REPORT 1

In 2002, a 76-year-old man presented with a PSA level of 15 ng/mL. His physical examination was normal, and a digital rectal examination revealed a slightly enlarged prostate. Biopsies revealed a prostate cancer with no evidence of distant metastases and a Gleason score of 7 (4 + 3). Given the patient's advanced age, surgery was excluded. Moreover, the patient refused to undergo radiotherapy; therefore, he was treated with leuprolide and oral bicalutamide.

Follow-up visits were scheduled every 3 months that included serial physical examination, routine blood evaluations that included serum PSA determinations at regular intervals (every 2–3 months) and radiological assessments every 6 to 12 months or as clinically indicated.

In January 2010, an episode of hematuria, urinary retention, and bilateral hydronephrosis with acute renal failure (creatinine 6.39 mg/dL) occurred, and a percutaneous ureterostomy was performed. However, although the episode of acute kidney failure was resolved, the patient presented with CKD in subsequent tests (creatinine 2.0 mg/dL, creatinine clearance 35 mL/min). Stage 3B CKD was diagnosed and required observation and control of blood pressure and other risk factors.

In May 2010, the patient reported low-grade, constant back pain. A radionuclide scintigraphy revealed several areas suggestive of metastatic bone disease (ie, the right parietal bone, lumbar spine, sternum, ribs, pelvis, and right femur), and a serum PSA determination revealed a level of 210 ng/mL.

Therefore, the patient began chemotherapy with estramustine phosphate 560 mg per os daily and zoledronic acid intravenously every 28 days. Laboratory data revealed the following values: haemoglobin level 11.8 g/mL, hematocrit 38.5%, white blood cell count (WBC) 4200 cells/mm^3^, platelets 179,000 cells/mL, blood urea nitrogen 69 mg/dL, creatinine 2.6 mg/dL, and creatinine clearance 26.92 mL/min (stage 4 CKD). Due to age of the patient, the normality of the electrolytes, and the low urea value, renal replacement was not considered. Abdomen CT revealed the presence of a bilateral percutaneous ureterostomy with reduction of both kidneys, particularly the left, and the CT also revealed the presence of multiple osteosclerotic lesions in the lumbar spine and pelvis.

**FIGURE 1:**
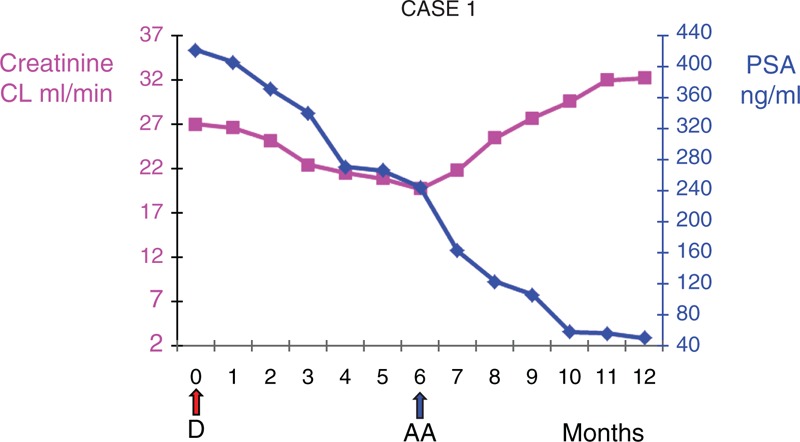
Trend of creatinine clearance and PSA during docetaxel and abiraterone acetate therapy.

Follow-up visits that included determination of the serum PSA were scheduled every 2 months. These evaluations revealed a progressive increase in PSA to 421 ng/mL (Figure 1); therefore, in July 2011, the patient received a second line of chemotherapy with docetaxel 40 mg/m^2^ intravenous infusion on days 1, 8, 15, 22, and 29 in a 6-week cycle.

Over the first 3 months, the PSA exhibited a small decrease to 339.7 ng/mL; after 6 months of treatment, the PSA went down to 243.7 ng/mL, but the laboratory data revealed a worsening of renal function with a blood urea nitrogen level of 60 mg/dL, a creatinine level of 3.5 mg/dL, and a creatinine clearance of 19.64 mL/min (stage 4 CKD). Furthermore, the patient reported a partial resolution of the back pain. In contrast, the patient experienced considerable toxicity with side effects including neutropenia, anemia, thrombocytopenia, fatigue, nail changes, dry eyes, which were grades I or II in most of the examinations, and a worsening of performance status. Treatment with docetaxel was discontinued, and, due to the toxicity, a hormonal approach with oral AA was undertaken (1000 mg die, administered as four 250-mg tablets and prednisone 10 mg die in 2 daily dosings).

Six months after beginning the new treatment, the PSA exhibited a substantial reduction (48.57 ng/mL; Figure 1), an improvement in renal function was observed (blood urea nitrogen 45 mg/dL, creatinine 2.1 mg/dL, and creatinine clearance 32.14 mL/min [stage 3B CKD]), electrolytes were normal, and the patient reported a reduction in bone pain with a decrease in analgesic consumption and an improvement in performance status. The treatment was well-tolerated without considerable side effects.

Currently, the patient is continuing treatment, his PSA level is 24.5 ng/mL (28 months without an increase in PSA), his creatinine is 2 mg/dL, and his creatinine clearance is 33.13 mL/min (stage 3B CKD).

## CASE REPORT 2

In November 2004, a 59-year-old man presented with a PSA of 13.7 ng/mL and a Gleason 7 (3 + 4) prostate adenocarcinoma. He underwent radical prostatectomy, and extensive perineural invasion and an extracapsular extension of the tumor were found. Subsequently, he was treated with radiation therapy and androgen-deprivation therapy consisting of leuprolide and oral bicalutamide.

Follow-up visits were scheduled every 3 months with serial physical examinations, routine blood evaluations, which included serum PSA determinations at regular intervals (every 2 to 3 months), and radiological assessments every 6 to 12 months or as clinically indicated.

**FIGURE 2:**
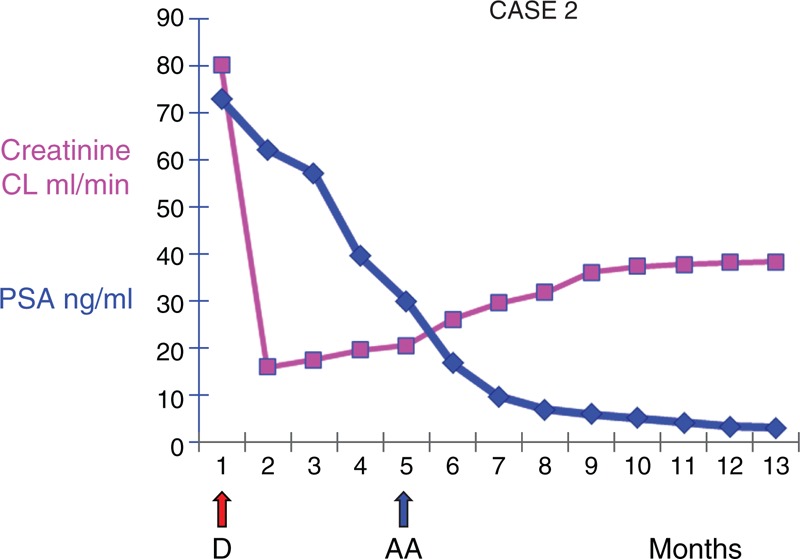
Trend of creatinine clearance and PSA during docetaxel and abiraterone acetate therapy.

In October 2009, his PSA increased to 53.45 ng/mL, and an abdominal magnetic resonance imaging identified metastases to the pelvic lymph nodes. From 2010 through 2011, the patient received docetaxel and epirubicin (11 cycles) followed by weekly navelbine per os for 6 months. In November 2011, there was a substantial increase in the PSA value (72.68 ng/mL; Figure 2), and the patient underwent docetaxel rechallenge with weekly docetaxel alone for 4 months.

During the first cycle of docetaxel rechallenge, an episode of urinary retention and bilateral hydronephrosis with acute renal failure (creatinine 7.34 mg/dL) occurred, and a percutaneous ureterostomy was performed. When the episode of acute kidney failure was resolved, laboratory tests revealed CDK with a creatinine level of 2.6 md/dL and a creatinine clearance of 24.62 mL/min. A stage 4 CDK was diagnosed and required observation and control of blood pressure and other risk factors. Other options were not considerate due to the age of the patient and the normality of the electrolytes and urea.

In March 2012, the PSA value decreased to 29.78 ng/mL; however, laboratory data revealed a partial improvement in renal function with a blood urea nitrogen level of 83 mg/dL, a creatinine level of 3.1 mg/dL, and a creatinine clearance of 20.32 mL/min (stage 4 CDK). Moreover, the patient began to suffer from the side effects of chemotherapy; therefore, some cycles were delayed due to toxicity.

Subsequently, he began AA (1000 mg die, administered as four 250-mg tablets and prednisone 10 mg die in 2 daily dosing). His PSA exhibited a substantial reduction (4.91 ng/mL; Figure 2), improvements in renal function were observed (blood urea nitrogen 57 mg/dL, creatinine 1.7 mg/dL, creatinine clearance 37.06 mL/min, stage 3B CKD), his electrolyte levels were normal, and the treatment was well tolerated without considerable side effects. Currently, the patient is continuing treatment, his PSA level is 0.1 ng/mL (24 months without an increase in PSA), his creatinine is 1.6 mg/dL, and his creatinine clearance is 38.16 mL/min (stage 3B CKD).

## DISCUSSION

Until recently, patients who had progressed following docetaxel treatment had very limited options that included mitoxantrone, which was the first cytotoxic agent to be approved for the palliative treatment of men with CRPC.^4^ Other agents have been tested, but most studies have reported no objective responses or responses of <15%,^5^ and unfortunately, all of these agents have provided short-term clinical responses that are associated with a considerable toxicities. Moreover, the antifungal agent ketoconazole, which is used as an inhibitor of the activities of several enzymes involved in androgen synthesis such as CYP17 and CYP11, has been investigated.^6^ However, ketoconazole lacks specificity for the CYP17 family of enzymes, which unfortunately leads to significant toxicities (eg, hepatotoxicity, gastrointestinal toxicity, and adrenal insufficiency).^6^

In the past 3 years, several new agents with different mechanisms of action have been developed for the treatment of prostate cancer^1,2,7,9–12^ with the goal of prolonging the overall survival of CRPC patients. Sipuleucel-T has been found to improve the overall survival of asymptomatic or mildly symptomatic men, most of whom had not received chemotherapy.^7^ However, sipuleucel-T has primarily been used in the United States and has been used less in Europe.^8^ Cabazitaxel was approved by the FDA for the treatment of patients with mCRPC who have previously been treated with docetaxel chemotherapy.^9^

Unfortunately, treatment with cabazitaxel is not without toxicity; indeed, severe neutropenia is common (89%), and 18% of patients discontinue the drug due to adverse events.^9^ Radium-223 has been reported to have a favorable safety profile with minimal myelotoxicity^10^; nevertheless, reports on the efficacy of radium-223 are recent, and at the time of this report, radium-223 was not commercially available. Rather, based on a systemic hormonal approach, AA and enzalutamide were approved for the treatment of patients with mCRPC following docetaxel treatment.^1,2–11^ Although enzalutamide exhibits a favorable toxicity profile,^11,12^ it could not be used for our patients because it is not yet commercially available; therefore, we opted for treatment with AA and excluded further lines of chemotherapy with cabazitaxel.

Since the first phase II studies of AA,^13^ it has been clear that the blockade of cytochrome CYP17 is associated with increased mineralocorticoid levels that result in some adverse events, the most common of which are hypokalemia, fluid retention, and hypertension; however, these toxicities are largely abrogated by the co-administration of low-dose glucocorticoids.

As illustrated by the 2 cases reported herein before, the patients continued to respond to treatment with docetaxel, but their laboratory data revealed worsening of their renal function that prevented the continuation of treatment with this chemotherapeutic agent. Conversely, after initiating AA, their renal functions exhibited improvements (Figures 1 and 2) without significant toxicity. Notably, in our patients, AA exhibited its efficacy via substantial PSA responses that were sustained over time. Indeed, after 28 and 24 months, the patient's PSAs did not increase. These durations are longer than the time to PSA progression and the overall median survival of patients who were treated with AA in the trial by de Bono (10.2 and 14.8 months, respectively). Interestingly, our patients also exhibited longer times to PSA progression than the median time of the patients in the COU-AA-302 trial (11.1 months). Therefore, the prognoses were better for our cases. However, it is difficult to explain the reasons for the prolonged survivals observed in our cases and whether the CDK played a role. To date, both patients are still receiving AA treatment.

## CONCLUSIONS

Recent studies showed that systemic hormone therapy is a well-tolerated option by the patients with CRPC. AA is one of the emerging novel therapies for advanced prostate cancer that employs a systemic hormonal approach.
